# What Next After Failed Septal Ventricular Tachycardia Ablation?

**DOI:** 10.1016/s0972-6292(16)30524-1

**Published:** 2012-07-28

**Authors:** Laurent Roten, Nicolas Derval, Patrizio Pascale, Pierre Jais, Pierre Coste, Frederic Sacher

**Affiliations:** 1 Service de Rythmologie, Hopital Cardiologique du Haut-Leveque and the Universite Victor Segalen Bordeaux II, Bordeaux, France.; 2 Soins Intensifs Cardiologiques - Plateau de Cardiologie Interventionnelle, Hopital Cardiologique du Haut-Leveque and the Universite Victor Segalen Bordeaux II, Bordeaux, France.

**Keywords:** ventricular tachycardia, bipolar radiofrequency ablation, ethanol ablation

## Abstract

Ablation of ventricular tachycardia (VT) by conventional radiofrequency ablation can be impossible if the ventricular wall at the targeted ablation site is very thick, as for example the ventricular septum. We present a case of a patient with incessant, non-sustained slow VT originating from the septal part of the lower outflow tracts. Radiofrequency catheter ablation from both ventricles as well as from the anterior cardiac vein were not successful. Both high power radiofrequency ablation and bipolar radiofrequency ablation neither were successfull. Finally, ethanol ablation of the first septal perforator successfully terminated arrhythmia. We discuss the possibilities to overcome failed conventional radiofrequency VT ablation of a septal focus.

## Introduction

Ablation of ventricular tachycardia (VT) originating from a septal focus can be impossible with radiofrequency energy because of insufficient deep tissue heating. We describe a case with a focal septal VT where this limitation of radiofrequency ablation was successfully overcome and discuss the alternative possibilities to conventional radiofrequency ablation.

## Case Report

A 64-years old patient with anterior myocardial infarction revascularized with CABG 16 years ago was implanted a cardioverter defibrillator (ICD) for primary prevention in 2005 because of impaired left ventricular ejection fraction (30%). In 2008 he experienced appropriate ICD interventions for VT. These VTs were successfully treated by amiodarone, which later had to be stopped because of hyperthyroidism. In the last years, he did well, was in functional class NYHA II and without ICD interventions on standard therapy including ACE inhibitor and betablocker.

**Figure 1:**
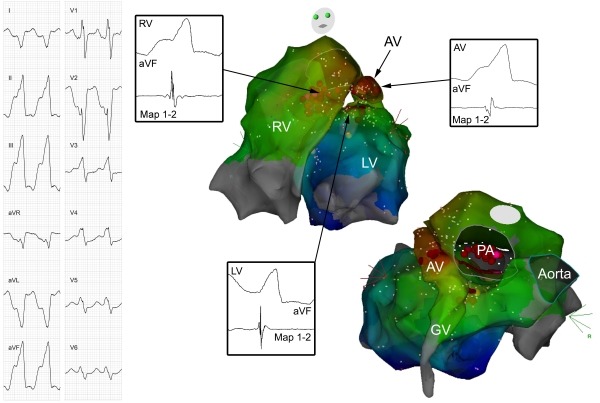
On the left side, the 12-lead ECG of the ventricular tachycardia is shown. On the right side, two views of an activation map of the VT are presented. Activation during VT in both ventricles as well as in the great cardiac vein (GV) and anterior cardiac vein (AV) is demonstrated. Recordings of distal ablation catheter (Map 1-2) and lead aVF at the earliest activation sites in the left ventricle (LV; 10 ms before QRS onset), in the right ventricle (RV, -20 ms) and in the AV (-20 ms) are shown. Brown dots represent ablation points, pink dots ablation points partially effective at higher power ablation. PA=pulmonary artery.

**Figure 2:**
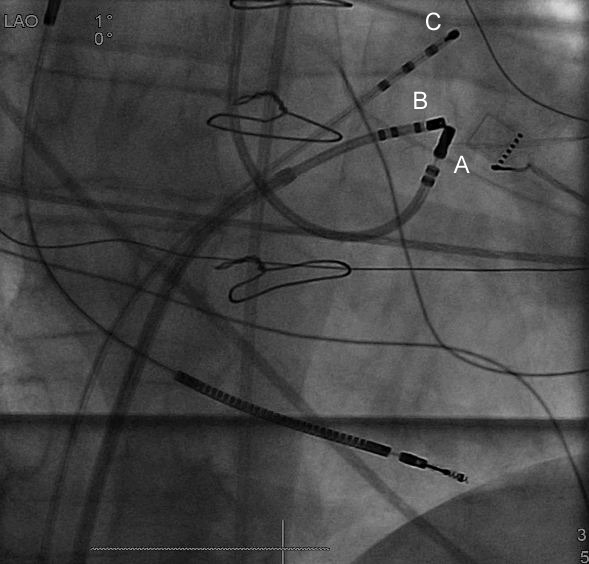
Antero-posterior fluoroscopic view of bipolar catheter ablation from the left and right ventricular outflow tracts. A first ablation catheter is positioned in the left ventricular outflow tract via a retrograde, aortic approach (catheter A) and a second ablation catheter in the right ventricular outflow tract via a long sheath (catheter B). Both catheters are placed at sites of earliest activation in the respective outflow tracts. A third mapping catheter is situated high in the right ventricular outflow tract (catheter C). Bipolar ablation was performed between the distal tips of both ablation catheters (A and B) with one catheter acting as cathode, and the other as anode.

The patient then developed incessant, mostly non-sustained, slow ventricular tachycardia (130 bpm) below programmed ICD intervention rate, associated with worsening functional class (NYHA III). Coronary angiography revealed open coronary artery bypasses and left ventricular ejection fraction was 40%. QRS morphology of the tachycardia was compatible with an origin in the left ventricular outflow tract (Figure 1) and ablation was attempted. Left and right ventricular sites activated first during tachycardia were located on the septal part of the lower outflow tracts of the respective ventricles, facing each other (Figure 1). On both ventricles, earliest activation preceded QRS onset (-10 ms in LV and -20 ms in RV). No scar tissue according to local electrogram amplitude was found on both sides of the septum. Ablation was attempted in the right and left ventricular outflow tracts without success (3.5 mm irrigated tip, 35 watts). In the anterior cardiac vein, earliest activation also preceded QRS onset by 20 ms. This site was interposed superiorly to the earliest sites in the left and right ventricular outflow tracts. Ablation with 20 watts in the anterior cardiac vein neither influenced tachycardia. Ablation with higher power was then attempted at the earliest site in the right ventricular outflow tract (60 watts, catheter irrigation at 60 ml/min.). This temporarily suppressed VT during ablation, but upon termination of ablation tachycardia recurred immediately. Two ablation catheters (one irrigated and one non-irrigated) were placed to face each other, one in the left and the other one in the right ventricular outflow tract at earliest activation sites (Figure 2). Bipolar ablation between the distal tips of both ablation catheters (with one catheter acting as cathode, and the other as anode) was performed without success. The procedure was then terminated with the patient still having incessant VT. Interestingly, VT ceased completely 4 hours after the procedure, but recurred 3 days later.

**Figure 3:**
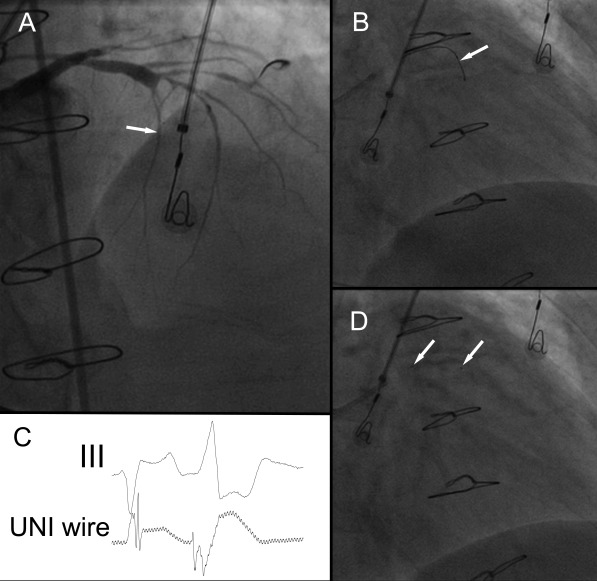
Ethanol ablation of the first septal perforator. A: Angiogram of the left coronary arteries (RAO 45º view). The left anterior descending coronary artery is diseased and stenotic, a first septal perforator is opacified (white arrow). B: A guidewire (white arrow) is introduced into the proximal part of the first septal perforator (RAO cranial view). C: Unipolar recording from the guidewire and electrocardiogram of lead III. The guidewire during this recording was in the position as shown in picture B. Earliest activation at this site preceded QRS onset by minus 50 ms. D: A balloon is introduced into the very proximal part of the first septal perforator (white arrows denote margins of balloon), and 1.5 ml of ethanol infused into the first septal perforator, successfully eliminating VT (RAO cranial view).

Because of recurrence of VT and the presumed origin in the proximal septum it was decided to attempt transcoronary ethanol ablation.[1,2] The coronary angiogram showed a prominent first septal perforator just before the stenotic part of the left anterior descending coronary artery. A guidewire was inserted into the first septal perforator and a unipolar electrogram recorded from the guidewire (Figure 3) as described by Segal et al.[3] In the proximal part of the septal perforator, earliest activity before QRS onset during tachycardia was minus 50 ms, and more delayed when advancing the guidewire deeper into the artery. The ostium of the septal perforator was occluded by a balloon and cold saline infused into the artery as described earlier.[1,2] This temporarily suppressed tachycardia. A total of 1.5 ml of absolute ethanol (96%) was then infused into the first septal perforator, upon which tachycardia ceased completely. Angiography showed a completely thrombosed first septal perforator. Troponin I rised to a maximum of 30 ng/ml (norm 0.00-0.04 ng/ml) two days after the procedure. After 2 months, the patient did not experience recurrence of ventricular tachycardia with improvement of functional class (NYHA II).

## Discussion

Ventricular tachycardia in patients with coronary artery disease is usually scar-related. In this case, despite reduced left ventricular ejection fraction and myocardial scar, tachycardia mechanism was probably focal as in classical forms of outflow tract VT. Both the absence of scar on electroanatomic mapping in the region of interest, as well as the repetitive, monomorphic pattern of VT point to this mechanism.

Electroanatomic mapping of earliest activation on both sides of the septum as well as in the anterior cardiac vein showed activation before QRS onset for each location, but nowhere activation was convincingly early. Therefore, and as can be seen in the 3D map (Figure 1), the origin of this tachycardia had to be in the basal, superior interventricular septum. Ablation from both ventricles and the anterior cardiac vein were not successful, probably because tissue heating by unipolar radiofrequency energy rapidly declines with tissue depth.

To overcome this limitation of unipolar radiofrequency energy, both power and catheter irrigation can be increased in order to have more effect on deeper tissue. This was temporarily successful in the present case, but the effect lasted only during radiofrequency application. Tissue heating obviously affected the focus, but was insufficient for coagulation necrosis at the site of tachycardia origin.

Another possibility to achieve deep tissue heating is bipolar radiofrequency ablation by two catheters facing each other on both sides of the tissue and connected by a custom switch box allowing bipolar ablation between the distal electrodes of both catheters (with one catheter acting as cathode and the other as anode). Bipolar radiofrequency catheter ablation has been shown to result in more transmural lesions and being less dependent on tissue contact.[4] Nevertheless, simultaneous alignment of both catheters is challenging, and current CARTO 3D mapping systems do only support visualization of a single ablation catheter. The attempt of bipolar radiofrequency ablation was neither successful in the case described. Eventually, bipolar radiofrequency ablation between the right ventricular outflow tract and the anterior cardiac vein, or the use of two irrigated catheters as well as higher power would have been successful, but this would also have increased the risk of side effects.

Finally, transcoronary ethanol ablation is another possibility to ablate deep intramyocardial foci and has been shown to be effective for septal ventricular tachycardias.[1,2] Alcohol septal ablation of the first septal perforator has been done for more than one decade for hypertrophic obstructive cardiomyopathy with reasonable safety and efficacy.[5] With an angioplasty wire, it is possible to record an unipolar electrogram from within the myocardium and to select the vessel in close proximity to the area of tachycardia origin.[3] In the present case, earliest activity in the proximal part of the first septal perforator was much earlier than any of the potentials recorded during the first procedure. Before infusion of ethanol and irreversible vessel damage, the effect of ethanol ablation can be temporarily simulated by infusion of iced saline or antiarrhythmic drugs.[1] To prevent spill over of ethanol into the left anterior descending coronary artery with unanticipated myocardial damage it is important to occlude the ostium of the targeted vessel by an inflated balloon. Also, collaterals from the first septal perforator to the right coronary arteries should be excluded before ethanol infusion. One important side effect of ethanol infusion is impairment of atrioventricular conduction with bundle branch block or complete atrioventricular block, necessitating permanent pacemaker implantation. As demonstrated in this case, ethanol septal ablation is an effective possibility to ablate deep septal foci when all alternatives have failed.
